# GENT2: an updated gene expression database for normal and tumor tissues

**DOI:** 10.1186/s12920-019-0514-7

**Published:** 2019-07-11

**Authors:** Seung-Jin Park, Byoung-Ha Yoon, Seon-Kyu Kim, Seon-Young Kim

**Affiliations:** 1 Genome Editing Research Center, Korea Research Institute of Bioscience and Biotechnology (KRIBB), Daejeon, 34141 Korea; 2 Department of Bioscience, University of Science and Technology (UST), Daejeon, 34113 Korea; 3 Personalized Genomic Medicine Research Center, KRIBB, Daejeon, 34141 Korea

**Keywords:** Tissue and cell line wide gene expression profiling, Cancer subtype profiling, Survival analysis, Meta-survival analysis, Large scale microarray web-database

## Abstract

**Background:**

Gene Expression database of Normal and Tumor tissues 2 (GENT2) is an updated version of GENT, which has provided a user-friendly search platform for gene expression patterns across different normal and tumor tissues compiled from public gene expression data sets.

**Results:**

We refactored GENT2 with recent technologies such as Apache Lucene indexing for fast search and Google Web Toolkit (GWT) framework for a user-friendly web interface. Now, GENT2 contains more than 68,000 samples and has several new useful functions. First, GENT2 now provides gene expression across 72 different tissues compared to 57 in GENT. Second, with increasing importance of tumor subtypes, GENT2 provides an option to study the differential expression and its prognostic significance based on tumor subtypes. Third, whenever available, GENT2 provides prognostic information of a gene of interest. Fourth, GENT2 provides a meta-analysis of survival information to provide users more reliable prognostic value of a gene of interest.

**Conclusions:**

In conclusion, with these significant improvements, GENT2 will continue to be a useful tool to a wide range of researchers. GENT2 is freely available at http://gent2.appex.kr.

**Electronic supplementary material:**

The online version of this article (10.1186/s12920-019-0514-7) contains supplementary material, which is available to authorized users.

## Background

The explosion of publicly available omics datasets including gene expression microarray and next-generation sequencing has provided valuable resources for researchers. However, for most researchers with few bioinformatics skills, public datasets in a raw data format are of little value. To help researchers to utilize public gene expression datasets, we previously developed a web-based gene expression database named Gene Expression database of Normal and Tumor tissues (GENT) [1]. GENT provides gene expression profiles across diverse human cancer and normal tissues with more than 34,000 samples generated by the Affymetrix U133A or U133Plus2 microarray platform with consistent data processing. To our satisfaction, since its launch at 2011, GENT has been widely used by researchers across the world, with over 1600 visitors per month on average, and the method has been cited more than 100 times in research work [2–6].

In our opinion, a few strengths have contributed to the success of GENT. First, it is very simple to use. A user inputs a gene symbol of interest and gets back results within a few seconds. Second, the large amounts of samples (~ 34,000) generated using the same platform with consistent data processing makes the database reliable by removing or reducing many biases (i.e., different batches, laboratories, ethnicities, etc.) caused by a small sample size. Third, GENT provides intuitive graphics as well as raw gene expression values to users, so users can utilize the information in convenient ways.

Enormous amounts of publicly available gene expression datasets have accumulated since GENT was first published in 2011, and the need was recognized to upgrade the GENT system both quantitatively and qualitatively. Here, GENT2 is provided as an updated version of GENT with more samples and newer functionalities. Major improvements of GENT2 over GENT are the following. First, GENT2 contains more than 68,000 samples compared to 34,000 samples in GENT. Second, GENT2 provides gene expression information on 72 paired tissues compared to 57 tissues of GENT. Third, GENT2 provides results of statistical tests (t-test, log2 fold changes, etc.) as well as boxplot summaries for user convenience. Fourth, considering the importance of tumor subtypes, GENT2 provides results of various cancer subtype profiling analysis (e.g., stage or grade with various molecular subtypes), Fifth, it provides results of prognostic value estimation using Kaplan-Meier plots. Finally, GENT2 provides results of meta-survival analysis combining various independent studies for reliable prognostic information. With these significant improvements, GENT2 is suggested as a versatile tool to assess the biological and prognostic relevance of a specific gene in various cancers.

## Construction and content

**Fig. 1:**
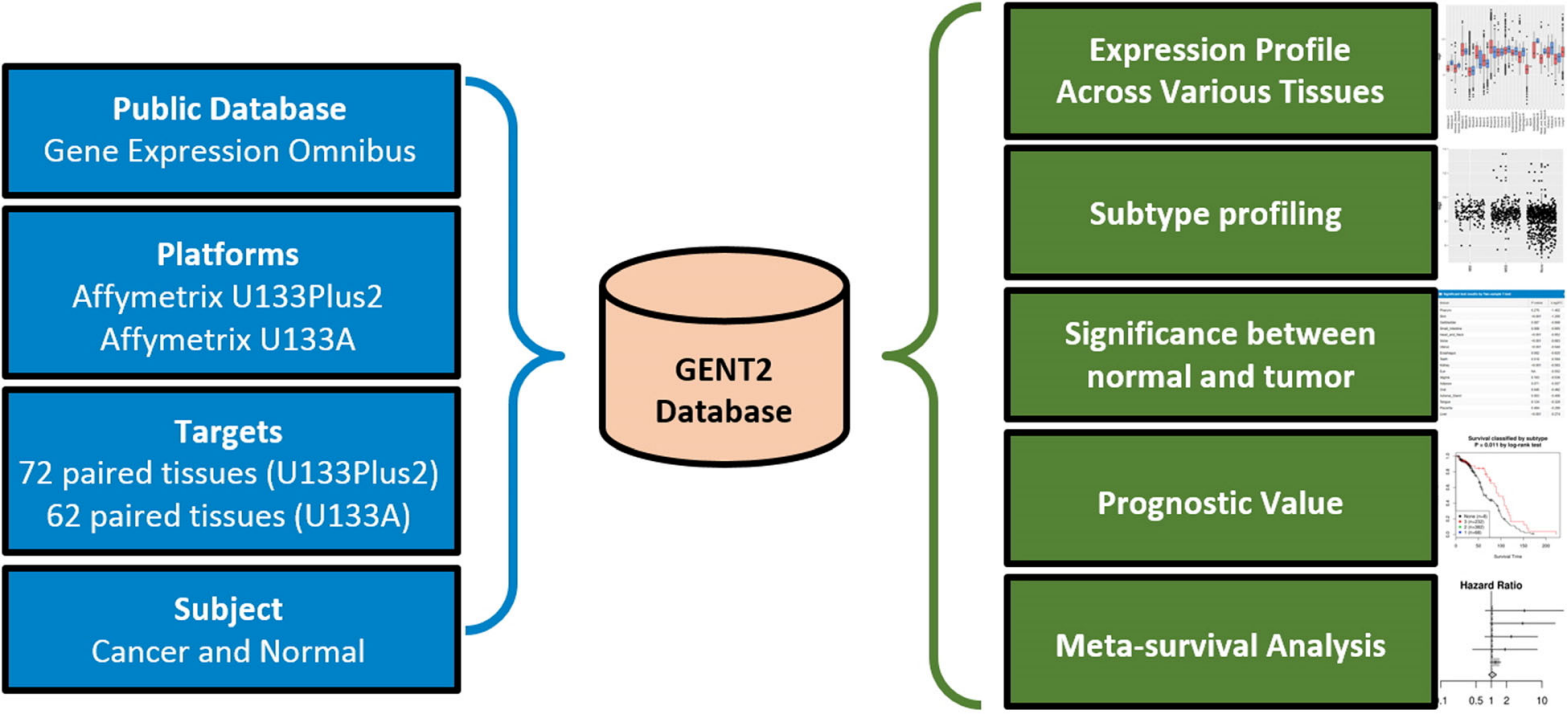
Overview of the GENT2 database system. We collected data from the GEO public repository using only two platforms, U133Plus2 (GPL570) and U133A (GPL96). Next, we classified the data into cancer and normal tissue. The functions of the GENT2 database are listed on the right

To construct the GENT2 system, gene expression data is downloaded from the NCBI GEO database generated by two microarray platforms (Affymetrix U133A or U133Plus2). Then, the data were stratified into cancer and normal samples across 72 tissues. The GENT2 database provides the following five functions: 1) a landscape of gene expression profile across 72 normal and tumor tissues, 2) cancer subtype profiling, 3) statistical significance of gene expression difference between normal and tumor samples, 4) a prognostic value of gene expression, and 5) meta-survival analysis (Fig. 1). The following sections present the workflows of how the database was constructed.

### Collection and processing of publicly available gene expression datasets

We collected data from the NCBI GEO public database and processed them with the MAS5 algorithm using the affy package in Bioconductor [7]. We used the same MAS5 algorithm used in the original GENT, as it is a single-array algorithm, which allows the comparison of multiple independent datasets [1].

### GENT2 implementation

The GENT2 system consists of the following two main parts: indexed DB and web-based user interface. To construct a database that can handle a huge gene expression profile (over 68,000 cancer patients) efficiently, we applied the Apache Lucene index machine (ver. 7.4.0) into whole gene expression datasets. A web-based graphical user interface (GUI) was implemented with the Google Web Toolkit (GWT, ver. 2.7.0) and GWT extended (GXT, ver. 4.0.0) frameworks based on JAVA language. Data exchange between a web browser and the GENT2 server is controlled by a GWT remote procedure call. All statistical tests provided from the GENT2, such as two-sample t-test, Kaplan-Meier with log-rank test, and meta-survival analysis with Cox proportional hazard modes, were implemented using R (ver. 3.2.5) with Bioconductor plugins (ver. 3.6). The system architecture of GENT2 is illustrated in Additional file 3: Figure S1.

### Database description

GENT2 contains more than 44,000 (U133Plus2; 887 datasets) and 23,000 (U133A; 358 datasets) samples (Table 1, Additional file 1: Table S1, Additional file 2: Table S2). A larger number of samples were added to the original GENT, and over 380 datasets were added for the U133Plus2 platform. The top three tissues of each platform are as follows: blood, brain and breast for U133Plus2 and blood, bone marrow and brain for U133A. Additionally, subtype analysis and meta-analysis data in GENT2 are displayed in Tables 2 and 3, respectively.

**Table 1:** GENT2 database description

	GENT			GENT2		
	U133Plus2 (GPL570)	U133A (GPL96)	Total	U133Plus2 (GPL570)	U133A (GPL96)	Total
Datasets	306	241	547	887	358	1245
Samples	24,300	16,400	40,700	44,857	23,283	68,140
Probes	54,613	22,215		54,613	22,215	

The GENT2 DB contains 887 (U133Plus2) and 358 (U133A) datasets, which contain more than 44,000 patient data records and more than 23,000 patient data records, respectively. The U133Plus2 platform is the most used in Gene Expression Omnibus (GEO)

**Table 2:** Subtypes of each cancer type

Tissue	Subtypes	Samples	Tissues	Subtypes	Samples
Adrenal Gland	Grade	29	Liver	Stage	80
Bladder	Stage Grade	93	Lung	Molecular Subtype Stage Histology	1074
Blood	Cancer Molecular Subtype Stage	5924	Oral	Stage	74
Brain	Cancer Grade Histology	2168	Ovary	Stage Grade Histology	940
Breast	Molecular Subtype ER status PR status HER2 status Grade Histology Histology Group	1246	Pancreas	Stage Grade	70
Cervix	Stage Histology	89	Prostate	Gleason Grade	46
Colon	Molecular Subtype AJCC Stage Duke Stage Grade Histology	1146	Skin	Stage	123
Endometrium	Stage Grade Histology	91	Stomach	Molecular Subtype TNM Stage Lauren Classification	300
Head and Neck	Stage	24	Thyroid	Cancer Stage	204
Kidney	Cancer Stage	182			
Total					13,903

**Table 3:** Datasets used with meta survival analysis in GENT2

Tissue	Prognosis Types			Datasets
	OS	PFS	RFS	
Adrenal Cortex	O			3
Bladder	O		O	4
Blood	O			5
Bone	O			5
Bone Marrow	O		O	16
Brain	O			11
Breast	O	O	O	53
Bronchus	O			1
Colon	O		O	20
Fibrous			O	1
Head And Neck			O	1
Kidney	O			1
Larynx			O	1
Liver	O			2
Lung	O	O	O	26
Lymph	O		O	4
Merkel Cell	O			1
Mouth(Tongue)	O			1
Myeloid Cell	O			8
Neural Crest	O			1
Ovary	O	O	O	20
Pancreas	O			2
Prostate	O			2
Sarcoma	O			1
Skin	O			3
Stomach	O			1
Thyroid	O			1
Total				195

*OS* Overall Survival, *PFS* Progression-Free Survival, *RFS* Recurrence-Free Survival

## Utility

### Tissue- and cell line-wide expression profile comparing normal and tumor samples

**Fig. 2:**
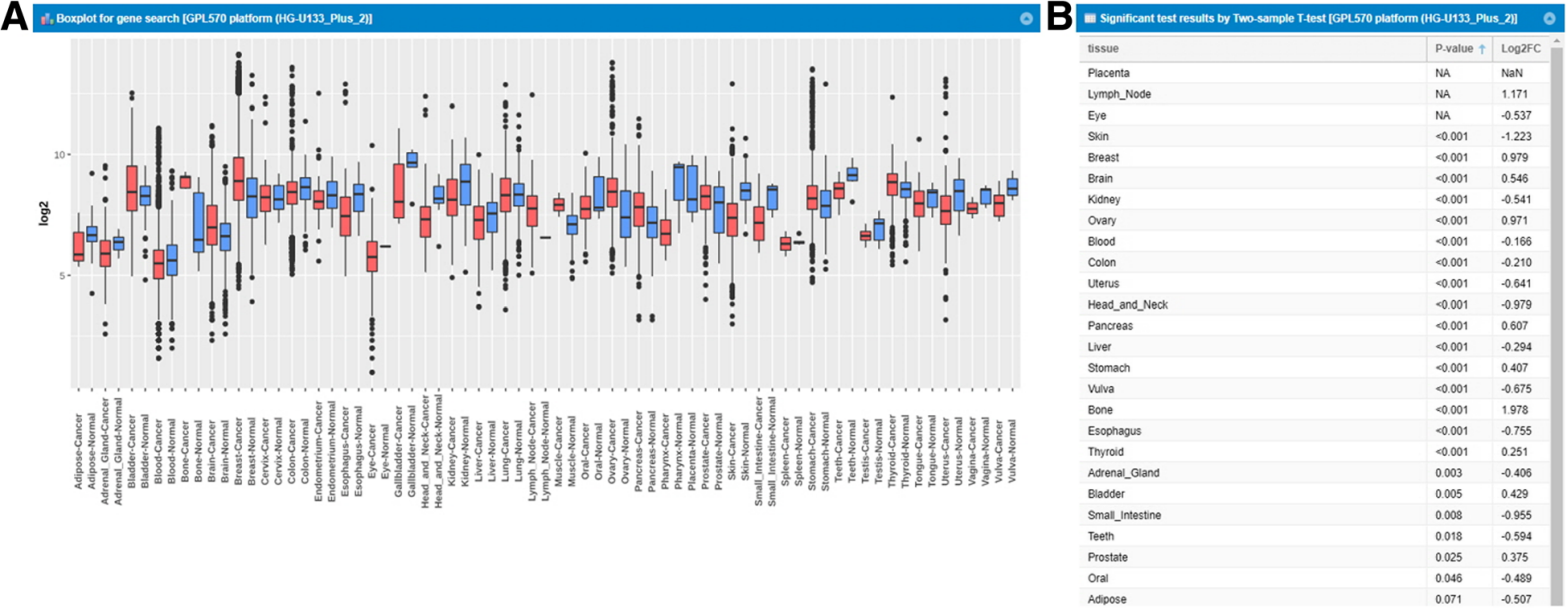
Tissue-wide gene expression pattern of ERBB2 gene across 72 paired tissues and statistical tests. **a** A boxplot across 72 paired tissues in GPL570 only. Red indicates a boxplot of cancer samples. Blue indicates a boxplot of normal samples. We exclude the tissue-wide boxplot of GPL96. **b** Statistical test based on expression profile at each tissue. For each tissue, *P-value* and log2 fold change are calculated, and NA at the ‘*P*-value’ column means that there is only one sample of normal tissue. Same above, the statistical test of GPL96 has been excluded

When a user puts a gene symbol in the ‘Search Gene profile’ page, GENT2 performs tissue-wide expression profiling and statistical testing quickly (less than 5 s) on two Affymetrix platforms, simultaneously. ‘Search Gene profile’ provides two boxplots of gene expression from the Affymetrix U133Plus2 and the Affymetrix U133A platform, allowing users to compare both results. In Fig. 2a, for example, we illustrate the tissue-wide gene expression profile of an oncogene, *ERBB2*. Compared to normal, *ERBB2* was overexpressed in a few tumors including from bone, breast, and ovary. The t-test (Fig. 2b) shows that there were significant expression differences between tumor and normal tissues in bone, breast and ovary tissues (3.9-fold and *P* < 0.001, 1.9-fold and *P* < 0.001, 1.9-fold and *P* < 0.001, respectively). *ERBB2* is a representative oncogene activated in breast cancer [8]. It was also reported that *ERBB2* overexpression helps metastatic progression of prostate cancer to bone [9]. Moreover, ovarian cancer was also linked to *ERBB2* activation, and many efforts are on-going to develop molecular cancer therapeutics by targeting the *ERBB2* pathway.

### Subtype profiling with survival analysis

**Fig. 3:**
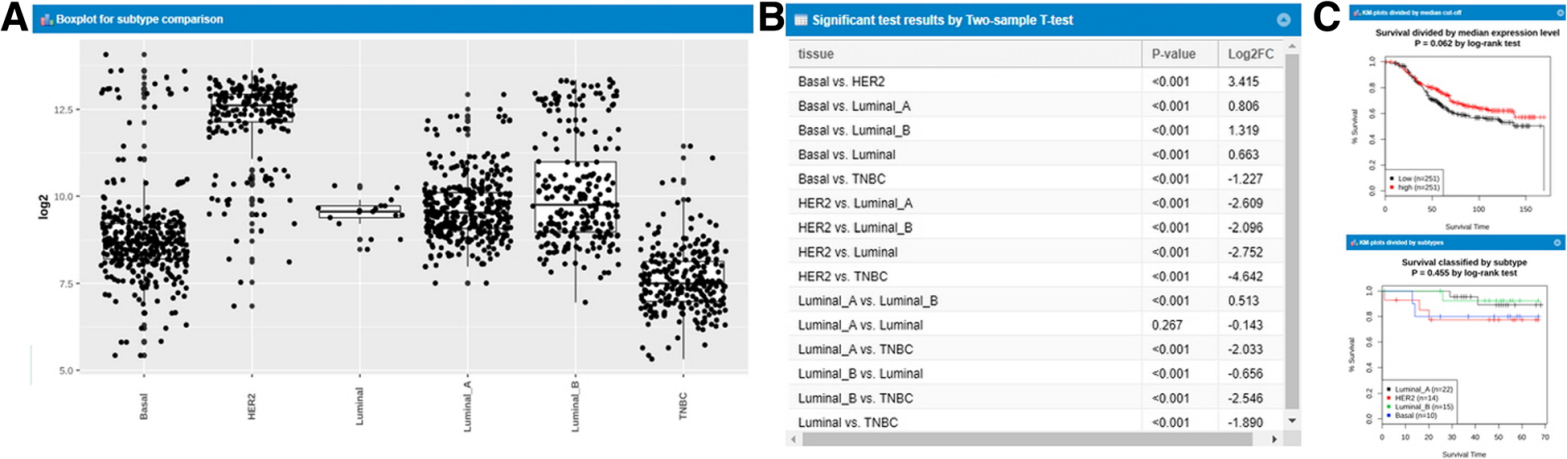
Subtype and survival analysis. **a** Box and dot-plot of each subtype. **b** Statistical test based on each subtype. For each subtype, *P-value* and log2 fold change are calculated. **c** Kaplan-Meier plots by subtypes and median cut-off

In GENT2, we added the following two novel functions: subtype profiling and survival analysis. If a user selects a tissue and a specific subtype, GENT2 provides the expression profiling of subtypes of the selected tissue. In addition, it provides results of a statistical test (ANOVA or t-test) and survival analysis on the subtype. For instance, the expression pattern for the molecular subtype of the ERBB2 gene in breast cancer tissue is shown in Fig. 3a. As expected, the HER2 group has the highest value on average, and the TNBC group has the lowest value. Statistical tests are also performed for each subtype (Fig. 3b). In particular, the ERBB2 gene has a highly distinct expression pattern with relatively all significant differences in each group. Finally, survival analysis is performed for each subtype (Fig. 3c). Although the ERBB2 gene showed distinct expression patterns, the log-rank test *p-value* for each group was found to be insignificant, suggesting that expression difference is irrelevant of prognostic difference. Currently, a subtype analysis function provides information on 46 subtypes of 19 tissue types with over 13,000 samples.

### Meta-survival analysis

**Fig. 4:**
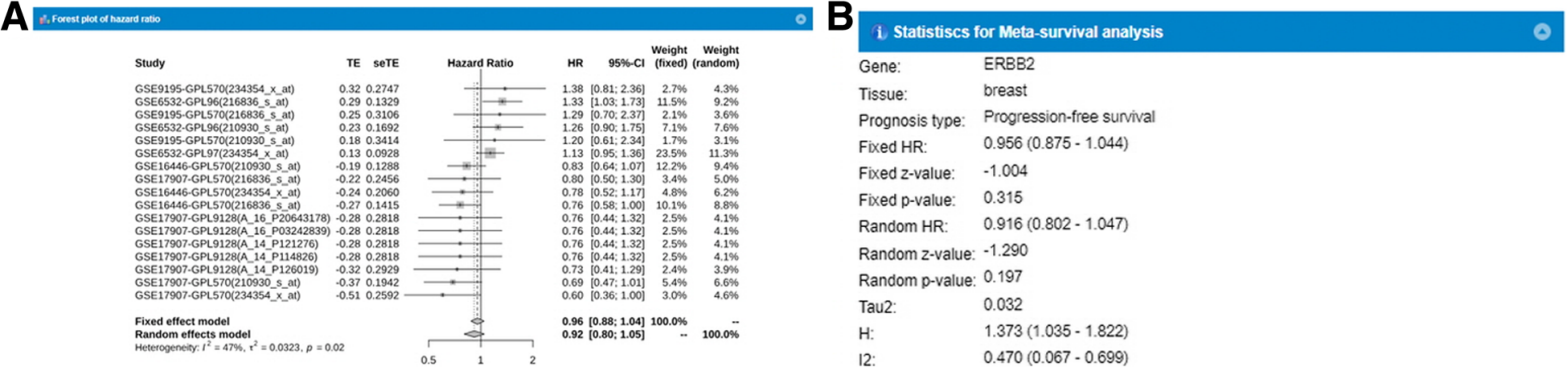
Meta-analysis. **a** Forest plot of hazard ratio on the ERBB2 gene with progression-free survival. Displays study name as well as GSE, GPL, and probe information. TE is the estimated treatment effect, seTE is the standard error of the treatment estimate, and HR indicates the hazard ratio average. Fixed and random effects are shown. **b** Summary and statistics test of **a**

Confirming the prognostic relevance of a gene is important for its application in the clinical field. Many studies have reported incongruent prognostic characteristics of a gene, thus there is a great need to further evaluate the prognostic potential of a gene by combining multiple information across many studies. Hence, we adopted a meta-survival analysis into GENT2, providing reliable prognostic power estimated by synergetic effect across many independent reports. This allows integrated statistical analysis from different studies, which greatly increases the number of samples and improves statistical power. For example, progression-free survival in breast tissue for the *ERBB2* gene was analyzed by meta-analysis (Fig. 4). The top six datasets indicate that the hazard ratios are over 1 for the same gene and the same tissue region (breast), while others show the hazard ratios under 1. This result suggests that the prognostic value of ERBB2 can be different according to different contexts. For example, the GSE9195 dataset is an experiment to identify the effect of tamoxifen in estrogen receptor positive (ER+) breast cancer [10], while the GSE16446 dataset is also an experiment to uncover the effect of anthracycline in estrogen receptor negative (ER-) breast cancer [11]. We suggest that comparing multiple datasets researchers can obtain more reliable prognostic information.

### Performance improvements by technological updates

In the previously constructed GENT, all search and display activities were implemented using PHP and MySQL environments. PHP is one of the widely used computational languages on the server side and MySQL is an open-source relational database management system that stores the large amounts of data. Upon exponentially increased gene expression data that are publicly available, there has been a great need to upgrade system architecture of the GENT to efficiently handle those huge data sets. Thus, we applied recent informatics technologies including GWT and Lucene into the GENT2. By using GWT framework, user-friendly and flexible web interfaces were equipped in the GENT2, and real-time interactions between web-client and server systems through GWT RPC method were also available. Especially, applying Lucene index machine, a high-performance information retrieval library, into the GENT2, ultra-fast access across enormous expression data from more than 68,000 samples is available. Compared with MySQL system, Lucene indexing in the GENT2 showed a better performance accessing faster than approximately 10 times.

## Discussion

**Fig. 5:**
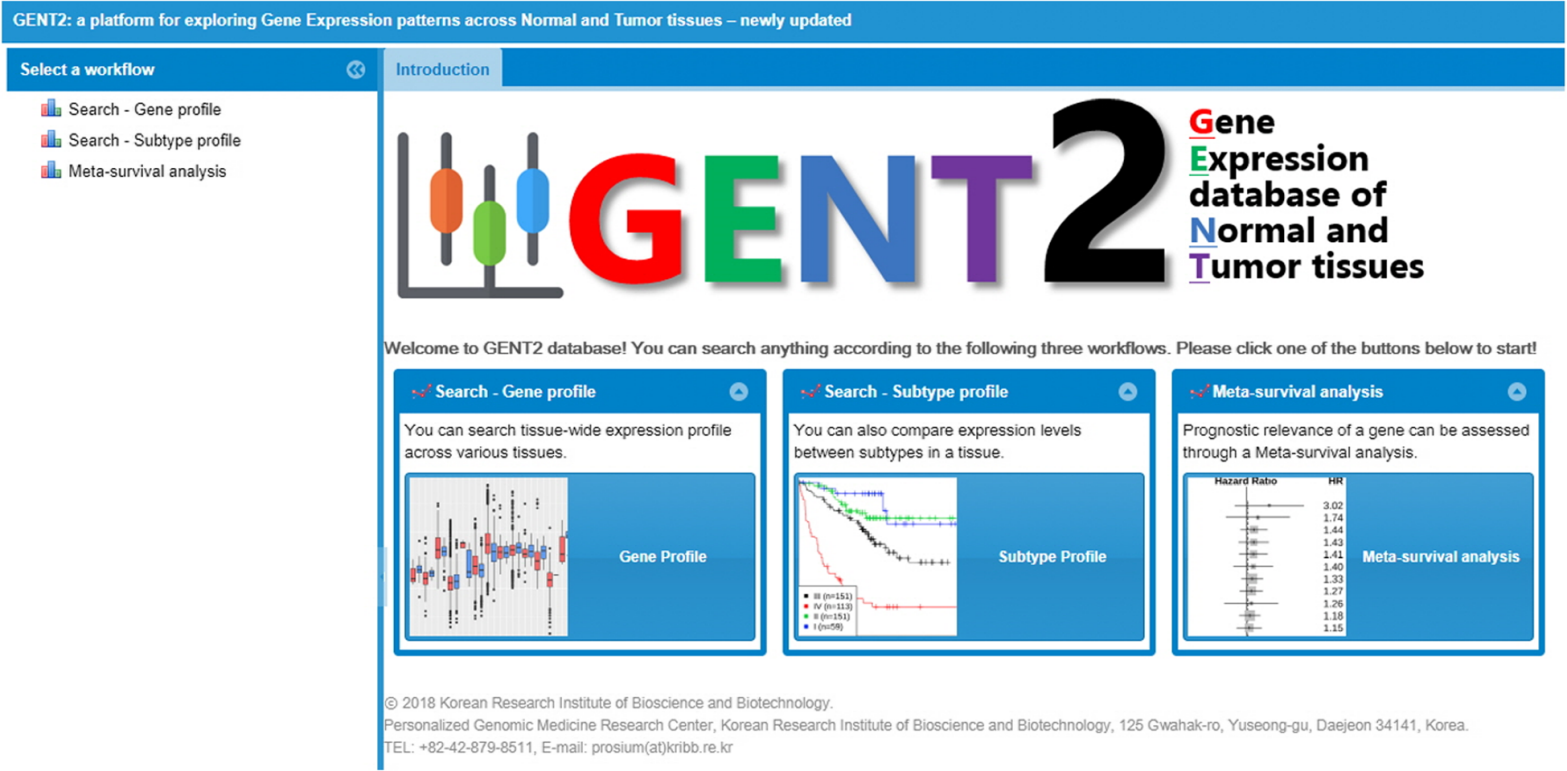
User Interface. The GENT2 main page consists of three layers for user convenience. First, the left layer is used to select three functions and can be hidden by clicking the arrow button. Next, the upper layer is a tab that allows users to select each window for different analyses. Finally, the center layer is a main window for displaying analysis results such as plots and tables

Many databases have been developed that show gene expression of cancer samples. For instance, Oncopression [12], CellLineNavigator [13], MERAV [14], Oncomine [15], cBioPortal [16], RNA Seq Atlas [17], KM-Express [18], BioXpress [19], TiGER [20], and so on (Table 4). Each database has its own unique advantages. First, Oncopression, CellLineNavigator, and MERAV are databases that can investigate gene profiling around the collected microarray datasets for various cancer types. Oncomine, cbioPortal, and RNA Seq Atlas are databases that analyze gene profiling for each dataset based on a microarray and RNA-sequencing. These databases have the benefit of comparing information between two high-throughput platforms. In addition, Oncomine and cBioPortal provide versatile and additional functions such as multidimensional comparison of samples (Oncomine) or integrated analysis of CNV, mutation, coexpression, and gene enrichment (cBioPortal). Lastly, the TiGER database contains EST data around several tissues. In many respects, they are very similar to GENT2 database. However, GENT2 has many useful features such as subtype analysis and meta-survival analysis, which are not available in other databases. In addition, an intuitive and user friendly interface is another advantage of GENT2 (Fig. 5). For example, the tab display in the GENT2 main page is useful when a user performs multiple iterations of analysis. In the near future, we plan to update GENT2 to enable multiple gene search and add RNA-sequencing data as well as microarrays.

**Table 4:** Comparison of GENT2 and other databases

Database	Data Type	Expression profiling			Cancer Subtype profiling	Meta-survival Analysis	REF
		Analysis Type	Cancer Type	The number of samples			
GENT2	Microarray	Aggregated	36	68,140	O	O	[1]
Oncopression	Microarray	Aggregated	20	19,000	X	X	[12]
CellLineNavigator	Microarray	Aggregated	28	317	X	X	[13]
MERAV	Microarray	Aggregated	27	4454	X	X	[14]
Oncomine	Microarray RNA-sequencing	Each	20	86,733	Various	X	[15]
cBioPortal	Microarray RNA-sequencing	Each	33	19,360	Various	X	[16]
RNA Seq Atlas	Microarray, RNA-sequencing	Aggregated	16	113	X	X	[17]
KM-Express	RNA-sequencing	Each	32	360	Various	X	[18]
BioXpress	RNA-sequencing	Each	23	6361	X	X	[19]
TiGER	expressed sequence tags	Aggregated	30	13,573	X	X	[20]

Aggregated; aggregated data presented

Each; each dataset presented

## Conclusions

The explosion of publicly available datasets in the cancer genomics field has provided cancer researchers invaluable resources. However, for many researchers who do not have bioinformatics skills to fully utilize the datasets, the public datasets are of little value. The widespread use of GENT since its launch at 2011 reflects the needs for carefully curated large-scale gene expression databases with an easy-to-use interface. With GENT2, we continue to provide the core functions of GENT; however, with increasing public datasets, we updated and expanded the GENT database in terms of datasets and novel useful functions. The number of different tissues increased from 57 to 72, and new functions such as subtype profiling, various statistical tests, and meta-survival analysis were added. We also adopted recent technologies such as such the GWT web framework and a Lucene indexing machine to provide more user-friendly web experiences. With those improvements in both data volume and novel functions, GENT2 will continue to be a useful tool to help researchers in the field of cancer genomics. As RNA-seq has become the de facto standard method for exploring gene expression, we plan to add gene expression datasets produced by RNA-seq in the future version of GENT.

## Additional files

Additional file 1: **Table S1.** U133Plus2 data description in GENT2. This is the data list for the U133Plus2 platform in the GENT2 database. For Blood, more than 10 K samples are stored. In particular, Blood, Breast, Colon, Kidney, Skin, and Bladder tissues contain all kinds of data. (PNG 16 kb)

Additional file 2: **Table S2.** U133A data description in GENT2. This is the data list for the U133A platform in the GENT2 database. There are no cell-line data from U133A platform around all tissues. (PNG 16 kb)

Additional file 3: **Figure S1.** GENT2 system architecture. The GENT2 infrastructure consists of web browser and server layers. A graphical user interface (GUI) was implemented using Google Web Toolkit (GWT) and GWT extended (GXT) frameworks. Transporting data between web browser and server is controlled by GWT remote procedure call (RPC) methods. All interactions in the server layer were implemented by R, RCaller, Apache Lucene software. (PNG 39 kb)
