# Supplementary material for: GENT2: an updated gene expression database for normal and tumor tissues
Source: BMC Med Genomics. 2019 Jul 11;12(Suppl 5):101. doi: 10.1186/s12920-019-0514-7 (PMC6624177; doi:10.1186/s12920-019-0514-7)
Supplement: Supplementary file 1 — Table S1. U133Plus2 data description in GENT2. This is the data list for the U133Plus2 platform in the GENT2 database. For Blood, more than 10 K samples are stored. In particular, Blood, Breast, Colon, Kidney, Skin, and Bladder tissues contain all kinds of data. (PNG 16 kb) [file 12920_2019_514_MOESM1_ESM.png]

**Table S1**. U133Plus2 data description in GENT2.

|  | **U133Plus2 (GPL570)** | | | | **Total** |
| --- | --- | --- | --- | --- | --- |
|  | **Tissue** | | **Cell-line** | |  |
|  | **Cancer** | **Normal** | **Cancer** | **Normal** |  |
| Blood | 13,198 | 1,097 | 618 | 30 | 14,943 |
| Breast | 5,574 | 475 | 643 | 10 | 6,702 |
| Brain | 3,439 | 873 | 242 | 0 | 4,554 |
| Colon | 3,775 | 397 | 332 | 2 | 4,506 |
| Lung | 2,362 | 508 | 530 | 0 | 3,400 |
| Ovary | 1,516 | 110 | 157 | 0 | 1,783 |
| Stomach | 1,028 | 117 | 132 | 0 | 1,277 |
| Kidney | 810 | 289 | 77 | 3 | 1,179 |
| Skin | 547 | 263 | 247 | 32 | 1,089 |
| Liver | 517 | 215 | 49 | 0 | 781 |
| Uterus | 432 | 58 | 102 | 0 | 592 |
| Thyroid | 320 | 171 | 18 | 0 | 509 |
| Pancreas | 324 | 105 | 79 | 0 | 508 |
| Prostate | 297 | 56 | 56 | 0 | 409 |
| Oral | 371 | 15 | 1 | 0 | 387 |
| Muscle | 3 | 355 | 10 | 0 | 368 |
| Esophagus | 236 | 24 | 46 | 0 | 306 |
| Bladder | 185 | 59 | 35 | 1 | 280 |
| Head_and_Neck | 217 | 20 | 23 | 0 | 260 |
| Eye | 156 | 1 | 9 | 0 | 166 |
| Cervix | 114 | 11 | 31 | 0 | 156 |
| Endometrium | 74 | 73 | 0 | 0 | 147 |
| Adrenal_Gland | 114 | 19 | 0 | 0 | 133 |
| Adipose | 10 | 72 | 0 | 0 | 82 |
| Lymph_Node | 55 | 1 | 0 | 0 | 56 |
| Tongue | 30 | 11 | 6 | 0 | 47 |
| Vulva | 21 | 14 | 9 | 0 | 44 |
| Teeth | 27 | 8 | 0 | 0 | 35 |
| Bone | 5 | 14 | 14 | 0 | 33 |
| Placenta | 0 | 17 | 9 | 0 | 26 |
| Pharynx | 15 | 3 | 7 | 0 | 25 |
| Small_Intestine | 12 | 11 | 0 | 0 | 23 |
| Gallbladder | 13 | 5 | 0 | 0 | 18 |
| Testis | 4 | 10 | 4 | 0 | 18 |
| Vagina | 3 | 5 | 0 | 0 | 8 |
| Spleen | 2 | 5 | 0 | 0 | 7 |
| Total | 35,806 | 5,487 | 3,486 | 78 | 44,857 |

This is the data list for the U133Plus2 platform in the GENT2 database. For Blood, more than 10 K samples are stored. In particular, Blood, Breast, Colon, Kidney, Skin, and Bladder tissues contain all kinds of data.
