# Supplementary material for: GENT2: an updated gene expression database for normal and tumor tissues
Source: BMC Med Genomics. 2019 Jul 11;12(Suppl 5):101. doi: 10.1186/s12920-019-0514-7 (PMC6624177; doi:10.1186/s12920-019-0514-7)
Supplement: Supplementary file 2 — Table S2. U133A data description in GENT2. This is the data list for the U133A platform in the GENT2 database. There are no cell-line data from U133A platform around all tissues. (PNG 16 kb) [file 12920_2019_514_MOESM2_ESM.png]

**Table S2**. U133A data description in GENT2.

|  | **U133A (GPL96)** | | | | **Total** |
| --- | --- | --- | --- | --- | --- |
|  | **Tissue** | | **Cell-line** | |  |
|  | **Cancer** | **Normal** | **Cancer** | **Normal** |  |
| Breast | 4,293 | 92 | 292 | 0 | 4,677 |
| Brain | 1,857 | 1,696 | 121 | 0 | 3,674 |
| Blood | 2,082 | 915 | 151 | 0 | 3,148 |
| Bone_Marrow | 2,390 | 81 | 70 | 0 | 2,541 |
| Lung | 1,117 | 499 | 414 | 0 | 2,030 |
| Colon | 1,112 | 127 | 80 | 0 | 1,319 |
| Kidney | 724 | 93 | 40 | 0 | 857 |
| Skin | 577 | 56 | 81 | 0 | 714 |
| Ovary | 605 | 22 | 49 | 0 | 676 |
| Stomach | 302 | 188 | 24 | 0 | 514 |
| Prostate | 323 | 86 | 23 | 0 | 432 |
| Muscle | 2 | 329 | 4 | 0 | 335 |
| Liver | 174 | 82 | 18 | 0 | 274 |
| Heart | 222 | 41 | 0 | 0 | 263 |
| Immune_System | 165 | 49 | 0 | 0 | 214 |
| Testis | 184 | 13 | 4 | 0 | 201 |
| Esophagus | 92 | 81 | 25 | 0 | 198 |
| Joint | 135 | 55 | 0 | 0 | 190 |
| Soft_Tissue | 149 | 9 | 19 | 0 | 177 |
| Urothelium | 90 | 9 | 20 | 0 | 119 |
| Uterus | 54 | 55 | 9 | 0 | 118 |
| Larynx | 111 | 2 | 0 | 0 | 113 |
| Thyroid | 59 | 28 | 13 | 0 | 100 |
| Pancreas | 41 | 14 | 18 | 0 | 73 |
| Adrenal_Gland | 58 | 7 | 2 | 0 | 67 |
| Bladder | 16 | 6 | 40 | 0 | 62 |
| Cartilage | 51 | 2 | 5 | 0 | 58 |
| Cervix | 31 | 10 | 15 | 0 | 56 |
| Tongue | 16 | 9 | 8 | 0 | 33 |
| Pharynx | 25 | 3 | 0 | 0 | 28 |
| Small_Intestine | 0 | 22 | 0 | 0 | 22 |
| total | 17,057 | 4,681 | 1,545 | 0 | 23,283 |

This is the data list for the U133A platform in the GENT2 database. There are no cell-line data from U133A platform around all tissues.
